# Transient equilibrium determination of dopamine D_2_/D_3_ receptor densities and affinities in brain

**DOI:** 10.3389/fnume.2022.1030387

**Published:** 2022-12-01

**Authors:** Jenny-Ann Phan, Dean F. Wong, Natalie H. S. Chang, Yoshitaka Kumakura, William R. Bauer, Albert Gjedde

**Affiliations:** ^1^ Department of Neurology, Gødstrup Hospital, Herning, Denmark; ^2^ NIDO - Centre for Research and Education, Gødstrup Hospital, Herning, Denmark; ^3^ Department of Neurology, Aarhus University, Aarhus, Denmark; ^4^ Department of Nuclear Medicine and PET Centre, Aarhus University Hospital, Aarhus, Denmark; ^5^ Johns Hopkins Medical Institutions, Department of Radiology and Radiological Sciences, Division of Nuclear Medicine PET Center, Baltimore MD, United States; ^6^ School of Medicine, Washington University in St. Louis, St. Louis, MO, United States; ^7^ Institute of Regional Health Research, University of Southern Denmark, Odense, Denmark; ^8^ Medical Spinal Research Unit, Spine Centre of Southern Denmark, University Hospital of Southern Denmark, Odense, Denmark; ^9^ Department of Diagnostic Radiology and Nuclear Medicine, Saitama Medical Center, Saitama Medical University, Moroyama, Japan; ^10^ Translational Neuropsychiatry Unit, Department of Clinical Research, Aarhus University, Aarhus, Denmark; ^11^ Departments of Nuclear Medicine and Clinical Research, University of Southern Denmark and Odense University Hospital, Odense, Denmark; ^12^ Department of Neuroscience, University of Copenhagen, Copenhagen, Denmark; ^13^ Department of Neurology and Neurosurgery, McGill University, Montréal, QC, Canada; ^14^ Neuroscience Center, Tabriz University of Medical Sciences, Tabriz, Iran

**Keywords:** receptor density, dopamine receptor, positron emission tomography, raclopride, bolus injection

## Abstract

Long-term alteration of dopaminergic neurotransmission is known to modulate the D_2_/D_3_ receptor expression in the brain. The modulation can occur as a response to pathological processes or pharmacological intervention. The receptor density can be monitored by *in vivo* positron emission tomography (PET) of [C11] raclopride. To obtain accurate measurements of receptor-ligand interaction, it is essential to estimate binding parameters at true (if transient) equilibrium of bound and unbound ligand quantities. We designed this study as a comparison of two quantitative approaches to transient equilibrium, the TRansient EquilibriuM BoLus Estimation (TREMBLE) method and the Transient Equilibrium Model (TEM) method, to determine binding parameters at transient equilibrium with bolus injection of the radioligand. The data demonstrates that TREMBLE unlike TEM identified the time at which equilibrium existed. TREMBLE revealed that equilibrium prevailed at one or more times after bolus injection and identified differences of receptor density among regions such as putamen and caudate nucleus. We demonstrated that TREMBLE is a quantitative approach suitable for the study of pathophysiological conditions of certain types of neurotransmission the brain.

## Highlights

1. We invented a method of quantitation of radioligand binding at transient but true equilibrium of binding (TREMBLE).
2. We compared the results of the new method of D_2_/D_3_ receptor density in striatum of humans with a standard method (TEM).
3. The method of TREMBLE yielded superior equilibrium binding estimates of the radioligand raclopride compared to TEM.

## Introduction

The D_2_/D_3_ dopaminergic receptor antagonist [C11] raclopride has been widely used to study the dopaminergic system by means of *in vivo* PET since 1985, when the tracer was introduced for the first time for human brain studies (1). The radioligand [C11] raclopride binds with high selectivity to $D_{2}$/$D_{3}$ receptors, and the binding is inhibited by challenge from endogenous dopamine (DA) (2,3) and other dopamine receptor agonists and antagonists (4). Thus, [C11] raclopride satisfies pharmacological criteria of application to studies of dopaminergic neurotransmission and receptor occupancy by drugs.

Dopamine mediates its physiological action through five subtypes of G protein-coupled receptors, D1-D5 with subtypes. Particularly, the $D_{2}$/$D_{3}$ receptors are highly expressed in brain areas critical for motor control, mesolimbic function, and memory processing, such as striatum, nucleus accumbens, olfactory tubercle, ventral tegmental area (VTA), and hippocampus [reviewed in Beaulieu and Gainetdinov (5)]. Therefore, dopaminergic signalling at these receptors is of special interest to neurological disorders with dysregulated dopaminergic transmission, including Parkinson’s disease (PD) and schizophrenia among others (6,7). Deficient dopaminergic transmission is one of the main underlying causes of motor impairment in PD, and post-mortem examinations have shown that the loss of dopamine is regionally heterogeneous, with greater loss in putamen than in caudate nucleus (8). Patients with PD display correspondingly greater $D_{2}$/$D_{3}$ receptor binding in putamen than in caudate nucleus, suggesting compensatory upregulation in response to dopamine deficiency (9).

Contrary to the dopaminergic deficits observed in Parkinson’s disease, increased dopaminergic activity is held to be an underlying pathophysiological characteristic of schizophrenia, as shown both by single-photon emission computed tomography (SPECT) and PET studies, in which amphetamine administration evokes greater dopamine release in patients with schizophrenia than in healthy control subjects (3,10–13). Interestingly, the magnitude of dopamine release in striatum evoked by amphetamine correlates significantly with changes of symptom severity on the Brief Psychiatric Rating Scale (BPRS) (3), and the rate of dopamine synthesis is higher in patients with schizophrenia than in healthy control subjects, as determined by means of PET with 3,4-dihydroxy-6-(18)F-fluoro-l-phenylalanine (FDOPA) (14).

To examine pathophysiology and effect of treatment by PET, it is necessary to estimate receptor density ($B_{max}$) and affinity ($1/K_{\text{D}}$) separately rather than binding potential. The necessity arises from the long-term modulation of dopaminergic neurotransmission that is known to lead to adaptive changes of the receptor system, as originally shown by early reports of autoradiography revealing increased $D_{2}$ receptor density in striatum by experimental administration of a selective $D_{2}$ antagonist (15,16).

The binding potential term originally introduced to PET quantification by Mintun et al. (17) as an equilibrium parameter reflects the ratio of bound to unbound radioligand that equals the product of the density of receptors without ligand and the affinity of the ligand ($B_{\max}^{\prime }K_{\text{D}}^{-1}$). Therefore, long-term modulation that alters the receptor density is reflected in the binding potential estimate. Of the several formulations of the binding potential, the term for the binding potential relative to non-displaceable binding in a region ($BP_{\text{ND}}$) was introduced by Innis et al. (18).

True receptor density and affinity must be determined at equilibrium with Eadie-Hofstee or Scatchard graphical analysis based on at least two levels of receptor occupancy. The gold standard of true albeit transient equilibrium in PET was achieved by continuous infusion of a radioligand that maintained a constant concentration in brain (19). However, continuous delivery of ligand experimentally is more difficult and any infusion conditions may not be more generalizable to all subjects in any given population than bolus injection approach. Therefore, quantitative kinetic models have been applied to bolus injection where the possible departure from the steady-state by continuous infusions are not an experimental limitation. The TRansient EquilibriuM BoLus Estimation (TREMBLE) method is an approach that yields the quantity of specifically bound radioligand on the assumption that the bound ligand reaches an equilibrium state at specific time points after bolus injection (20,21). The approach uses the radiolabelled metabolite-corrected plasma concentration of radioligand to specifically differentiate displaceable from non-displaceable receptor bound quantities of the radioligand, as derived in subsection I of the Appendix below.

To avoid the demand for arterial blood sampling, several approaches were presented in the past that apply an alternative reference region-derived input. One example is the Transient Equilibrium Model (TEM) that uses a reference region, e.g., the cerebellum, as an approximation of the tracer input from the activity in a region of non-displaceable binding (22), as derived in subsection II in the Appendix below. Both TREMBLE and TEM were designed to identify time points of transient equilibrium, as derived below in the Appendix. The aim of the present study was to compare the two approaches for quantification of $D_{2}/D_{3}$ receptor density using [C11] raclopride binding in healthy human brain.

## Material and methods

### Subjects

PET data of 21 healthy subjects were included in this study as a subset of a previous cohort (23). The criteria of selection of subjects for this study were based on the availability of data with blood sampling because the analysis with TREMBLE requires knowledge of tracer concentrations in arterial plasma. At the time of enrolment, all participants underwent physical examination, with no display of any abnormal neurological findings. The subjects had no neurological or psychiatric diseases in the past history, nor gave any evidence of substance abuse. All subjects signed informed consent forms prior to participation. The experiments were conducted at the Johns Hopkins Hospital in accordance with the Declaration of Helsinki as approved by the Institutional Review Board (IRB) of the Johns Hopkins Hospital.

**Figure A4:**
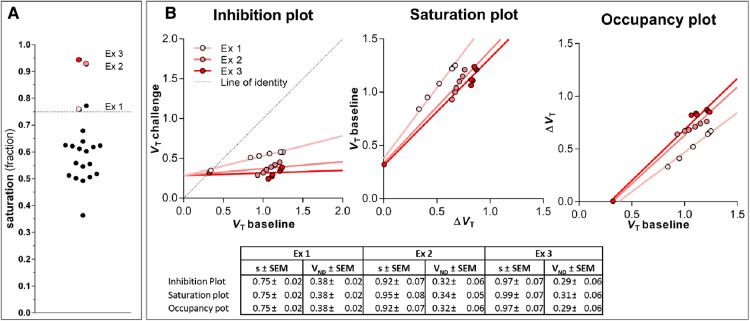
Magnitude of blocked receptors induced by challenge with unlabeled raclopride. Panel (**A**) shows a overview of the percentage of blocked receptors (s) induced by challenge in all subjects. The coloured points highlight the excluded subjects denoted as Ex 1-3. These particular subjects were excluded because their $m_{\text{b}}$ curves in the TREMBLE analysis were fluctuating around zero, leading to incorrect $BP_{\text{ND}}$ estimates. When the degree of blocked receptors exceeded 75%, the estimate of $B_{\text{max}}$ became unreliable because of inaccurate $BP_{\text{ND}}$ values. The dashed line in (**A**) indicates the threshold separating outliers due to excessive receptor blocking. Panel (**B**) illustrate the three analogous plots, which are designed to determine receptor occupancy, including Inhibition (51,52), Saturation (53) and Occupancy (54) plots, as recently summarized by presented by Khodaii et al. (49). The table in (**B**) lists the estimates obtained from the plots. The fraction of blocked receptors is denoted s. $V_{\text{ND}}$ reflects the estimate of the non-displaceable distribution volume, which was identical with the measured volume of distribution in cerebellum.

Three subjects were excluded from the analysis because they displayed maximal receptor blockade at 75–97% in the challenge condition (as shown in subsection III in the Appendix with details of the basis of exclusion shown in Figure A4 and listed in Table A1). Complete or close-to-complete receptor blockade is not applicable to estimation of receptor density, because the Eadie-Hofstee linearisation requires evidence of receptor occupancy at a minimum of two different degrees of occupancy. Therefore, the remaining 18 subjects of the original 21 subjects had an average age of 35±14 (mean±SD). The eleven women and seven men included subjects of Caucasian (13), African-American (4), and Asian (1) ethnicity.

### PET acquisition and analysis

All subjects underwent dual [C11] raclopride PET acquisitions at baseline followed by challenge with unlabeled raclopride. Molar activities were 359±432 GBq/μmol (mean±SD) and 0.67±0.14 GBq/μmol, respectively. Individual values are also listed in Table A2. Each acquisition lasted 90 min, during which we drew arterial blood samples to determine tracer input concentrations.

**Figure 1:**
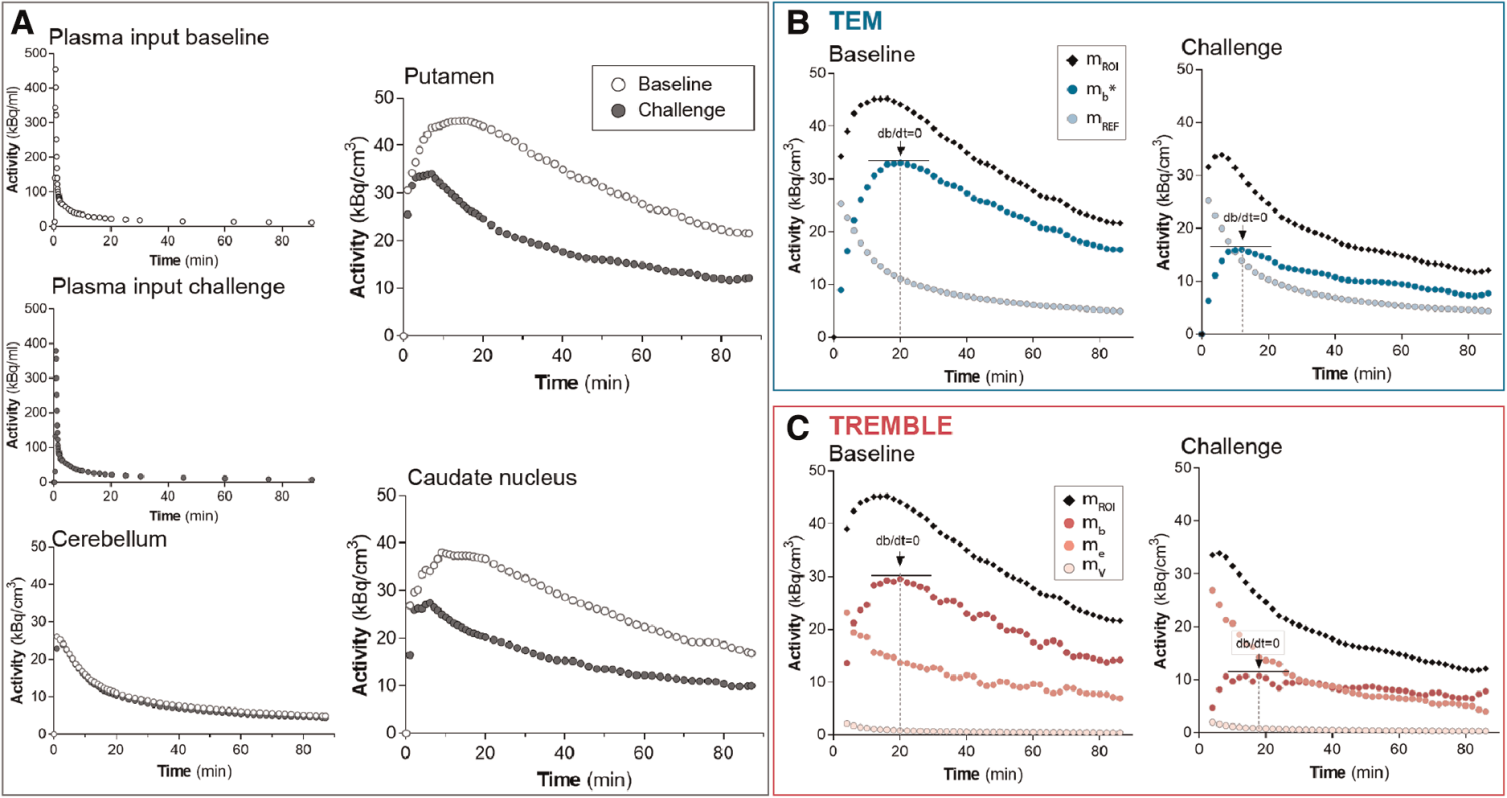
Time-activity curves from a representative subject. The graphs in panel (**A**) show the measured activities. The plasma input and cerebellum activities of challenge conditions were comparable to those at baseline. The activity in putamen and caudate nucleus declined markedly in response to challenge with non-radiolabeled ligand. Panels (**B**) and (**C**) present the computed activities in putamen based on the compartment models TEM and TREMBLE, respectively. The arrows in panels (**B**) and (**C**) indicate the times of transient equilibrium ($dm_{\text{b}}/dt=0$).

We analyzed the data by two kinetic models, described in subsections I and II in the Appendix, using a GUI custom-built in MATLAB (Mathworks), available at MATLAB central (24). Both models were designed to identify the instances of true but transient equilibrium of the bound quantity $m_{\text{b}}$ of tracer as the time(s) at which $dm_{\text{b}}/dt=0$, e.g., at the peak of the binding curve (as indicated with arrows in Figures 1B,C). We automatically identified The time of steady-state by means of a function that pinpointed the numeric maximum of an array. A few subjects displayed a flat $m_{\text{b}}$ curve in the challenge condition of the TREMBLE analysis. In those cases, we determined the bound quantity as the mean value in the interval 20–60 min of the $m_{\text{b}}$ curve.

### Statistics

To test if $B_{\text{max}}$ and $K_{\text{D}}$ estimates obtained by TREMBLE differed from the stimates obtained by TEM, we did one-way ANOVA, followed by Tukey’s correction of multiple comparisons. Paired t-tests were completed to test whether the $BP_{\text{ND}}$ estimates of the challenge condition differed from baseline. The applied statistical methods are specified in the respective figure legends. For all tests, we considered a P-value of less than 0.05 to be indicative of significance. Statistical tests were performed by Graphpad Prism v. 7.00.

### Compliance with ethical standards

The authors have no conflict of interest. Twenty-one healthy human participants were enrolled in the study, of whom 18 met the present criteria of moderate degrees of receptor occupation (i.e., 75% or less) by unlabeled raclopride. All subjects signed informed consent forms prior to participation. The experiments were conducted at Johns Hopkins Hospital in accordance with the Declaration of Helsinki, as approved by the Institutional Review Board (IRB) of the Johns Hopkins Hospital.

## Results

The time-activity curves from a representative healthy subject demonstrate that accumulation of [C11] raclopride was markedly reduced upon challenge with unlabeled ligand in both putamen and caudate nucleus when compared to the baseline condition (Figure 1A). The magnitude and profile of the plasma input concentrations were similar in the two conditions, in support of the interpretation that the lower tracer binding in the challenge conditions in target regions of dopaminergic neurotransmission was due to competition from unlabeled ligand and not from differences of the arterial input concentrations. Cerebellum, known to be devoid of dopamine binding sites, had identical time-activity curves in the two conditions, indicating that no displacement of bound ligand occurred in the reference region.

The binding curves obtained by the TREMBLE and TEM analyses of this representative subject are shown in Figures 1B and 1C, respectively. For the TEM analysis, we used the activity of the reference region ($m_{\text{REF}}$) as an approximation of the unbound and non-displaceable tracer quantity in brain. For the TREMBLE analysis, we computed the equivalent exchangeable quantity ($m_{\text{e}}$) from the measured arterial plasma input concentrations. The computed curve of $m_{\text{e}}$ initially was higher in the challenge condition than at baseline, suggesting a greater exchangeable quantity of [C11] raclopride as a result of displacement by unlabeled raclopride. This phenomenon was not observed with the TEM analysis.

**Figure 2:**
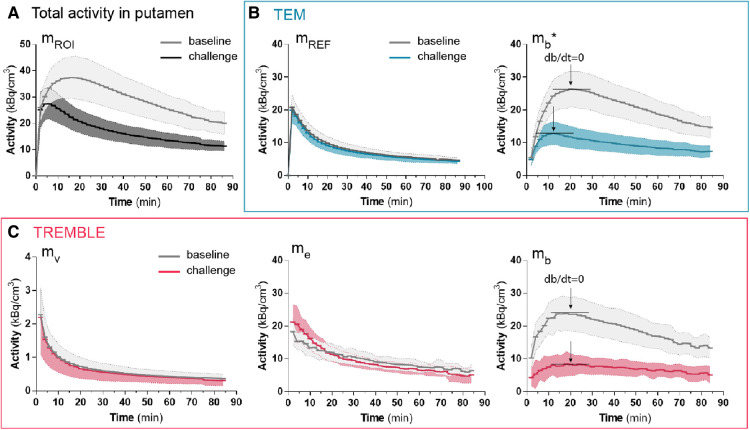
Population averaged time-activities in specific compartments of the putamen. The measured total activity in putamen is shown in (**A**). Panel (**B**) and (**C**) show the activities computed by TEM and TREMBLE, respectively. The arrows indicate the time of transient equilibrium (db/dt=0). The bound quantity as calculated with TEM is denoted with a star ($m_{\text{b}}^{*}$), whereas $m_{\text{b}}$ without annotation reflects the bound quantity as computed with TREMBLE. Notably, the magnitude of exchangeable quantity ($m_{\text{e}}$) was initially greater at challenge condition compared to baseline (panel (**C**) middle). For all the curves, the solid lines show the population mean and the shaded areas indicate the standard deviation.

The population average of the contents of respective compartments in putamen are shown in Figure 2. The population averaged curves consistently displayed the same time course pattern as curves of the single representative subject. In the challenge condition, at 0–20 min, the accumulation of ligand in the exchangeable compartment ($m_{\text{e}}$) exceeded that of the baseline condition, after which time the $m_{\text{e}}$ curves approached similar levels in both conditions (Figure 2C). We considered the initially higher $m_{\text{e}}$ quantity during the challenge condition a result of the lower receptor availability of receptors blocked by unlabeled ligand. This behavior of the exchangeable quantity was detected by the TREMBLE analysis but not by the TEM analysis.

**Figure 3:**
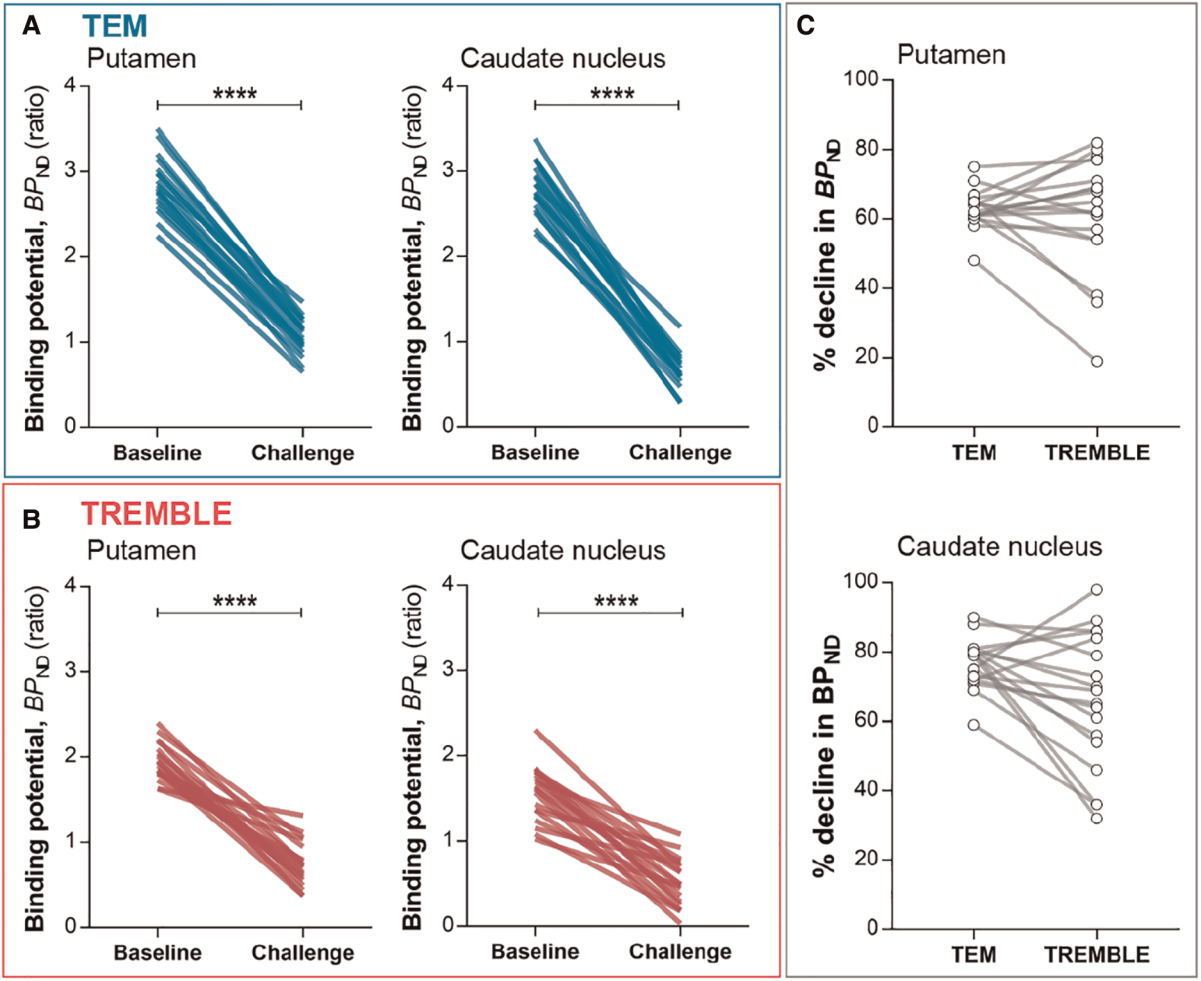
Binding potentials. $BP_{\text{ND}}$ in putamen and caudate nucleus declined significantly upon challenge as determined by TEM (**A**) and TREMBLE (**B**). Panel (**C**) presents the individual percentage decreases in $BP_{\text{ND}}$ in response to challenge as determined by the two models. Each line connects a pair of measurements in the same subject. Two-tailed paired t-test, p<0.0001.

Challenge with non-radiolabeled raclopride significantly reduced the estimates of $BP_{\text{ND}}$ by the two methods (Figure 3). The average reductions of the $BP_{\text{ND}}$ estimates averaged 61±17% and 63±6% (mean±SD) in putamen by TREMBLE and TEM, respectively. As the two models yielded similar reductions of $BP_{\text{ND}}$, we asked whether a scalable difference existed between the results of the two methods. We compared the percentage declines in each subject by TREMBLE and TEM (Figure 3C) and found no consistent pattern, implying that the outcomes of TREMBLE and TEM yield random differences.

**Figure 4:**
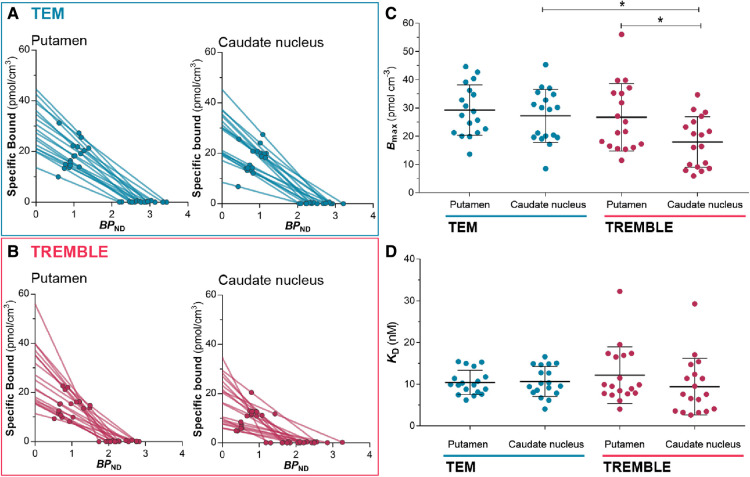
Estimates from Eadie-Hofstee plots. Eadie-Hofstee plots of putamen and caudate nucleus computed by TEM (**A**) and by TREMBLE (**B**). The receptor density (**C**) was obtained from the y-intercept, and $K_{\text{D}}$ (**D**) was determined from the slope on the respective Eadie-Hofstee plots. The receptor density was significantly lower in caudate nucleus than in putamen but this was only evident through TREMBLE analysis. There was a significant difference between $B_{\text{max}}$ estimated by TEM compared to TREMBLE. One-way ANOVA followed by Tukey’s multiple comparison test, *p<0.05.

With the estimates of specific binding ($M_{b}$, $M_{b}^{*}$) and binding potential ($BP_{\text{ND}}$) at two occupancy levels in the absence and presence of unlabelled raclopride, we applied Eadie-Hofstee plots to obtain the receptor density, $B_{\text{max}}$, and half-saturation constant, $K_{\text{D}}$, as presented in Figures 4A,B and subsection IV in the Appendix below. Comparison of receptor densities in Figure 4C revealed that TREMBLE detected a regional difference of $B_{\text{max}}$ with significantly higher density in putamen (26.7±11.9 pmol/${\mathrm{cm}}^{3}$) than in caudate nucleus (18±8.9 pmol/${\mathrm{cm}}^{3}$). In contrast, analysis with TEM yielded no difference of receptor density between the two parts of the striatum. Figure 4D shows the same values of $K_{\text{D}}$ in the two brain regions, regardless of analysis method.

**Figure 5:**
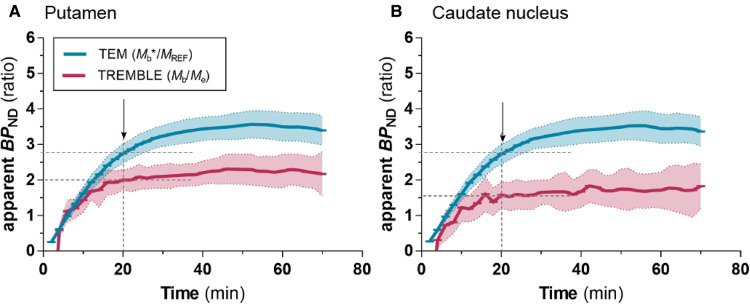
Apparent $BP_{\mathrm{ND}}$ values with TREMBLE and TEM. The blue curve shows estimates obtained with TEM analysis, and the red curve shows estimates from TREMBLE analysis. The solid curves show the mean and the shaded areas the standard deviation. The arrows indicate the time of which steady-state approached (at $dm_{\text{b}}/dt=0$). Notably, the time of steady-state coincided with the time, where $BP_{\text{ND}}$ approached a constant level as determined by TREMBLE.

We examined the dynamic time courses of the apparent $BP_{\text{ND}}$ estimates over time of both models, because the result of the Eadie-Hofstee plot reflects the magnitude of $BP_{\text{ND}}$. Interestingly, TREMBLE revealed that estimates of $BP_{\text{ND}}$ reached a plateau at approximately 20 minutes after bolus injection when $dm_{\text{b}}/dt=0$ (Figures 5A and B). This finding is consistent with the hypothesis that peaks or troughs identified by TREMBLE mark the times of true transient equilibrium. The apparent constant magnitudes of $BP_{\text{ND}}$ reflect the times when the ratios of bound-to-free ligand quantities are sustained for longer intervals. In contrast, the apparent values of $BP_{\text{ND}}$ continuously rose for 40 minutes when quantified by TEM.

**Figure 6:**
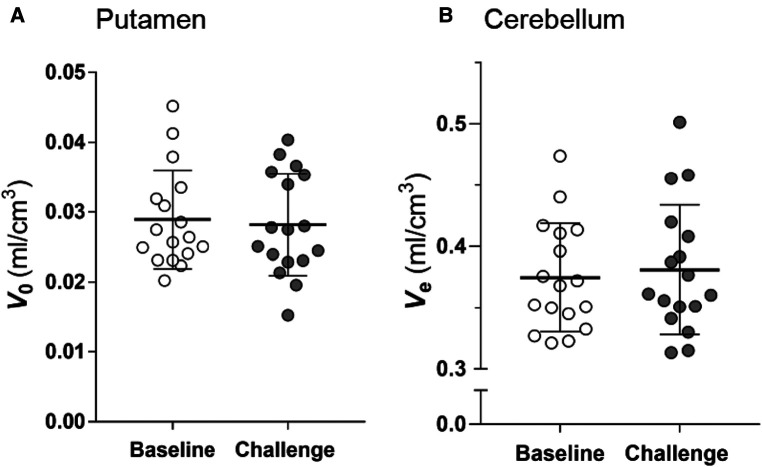
Estimates of vascular volume in brain ($V_{0}$) and distribution volume in cerebellum ($V_{e}$). The comparison of the $V_{0}$ and $V_{e}$ estimates at baseline and challenge condition shows that the estimates did not change in response to challenge. Two-tailed paired t-test.

Because the estimations of $m_{v}$ and $m_{e}$ by TREMBLE is influenced by the magnitudes of two constants, $V_{0}$ and $V_{e}$ (Eqs. A1 and A5), we examined if the constants were affected by challenge with unlabeled raclopride. As shown in Figures 6A and B, the constants were not affected by the unlabelled raclopride.

## Discussion

We determined $D_{2}$/$D_{3}$ dopamine receptor densities in putamen and caudate nucleus, using two different kinetic models, TREMBLE and TEM, to identify the binding at transient equilibrium. Although, the averages of $BP_{\text{ND}}$ decreased significantly upon blocking with unlabeled raclopride according to both methods, the individual estimates of $BP_{\text{ND}}$, $B_{\text{max}}$ and $K_{\text{D}}$ by the two models were not related.

Values of $B_{\text{max}}$ and $K_{\text{D}}$ obtained by the Eadie-Hofstee plot of TEM data match the results of previous PET studies that included TEM analysis (see Table 1 for a summary). In the present study, the TEM analysis yielded no significant difference of receptor density between the regions of putamen and caudate nucleus. In contrast, TREMBLE analysis of the same dataset showed a significantly greater receptor density of putamen than caudate nucleus.

**Table 1:** Comparison of $B_{\max}$ and $K_{D}$ of $D_{2}$/$D_{3}$ receptors with other [C11] raclopride studies in healthy human subjects %Diff denotes the percentage difference in $B_{\max}$ between putamen and caudate nucleus.

Brain region	n	Kinetic	$B_{\max}$	% Diff	$K_{D}$	Age	Reference
		model	(pmol/${\mathrm{cm}}^{3}$)		Mean (SD)	Mean (SD)	
			Mean (SD)				
Putamen	18	TEM	29.3 (8.9)	8	10.4 (2.9)	34.9 (14.0)	This study
Caudate nucleus		TEM	27.2 (9.4)		10.6 (3.6)		
Putamen		TREMBLE	26.7 (11.9)	48	12.2 (6.7)		
Caudate nucleus		TREMBLE	18 (8.9)		9.1 (6.7)		
Putamen L	20	TEM	27.7 (7.9)	25	8.9 (2.5)	27.5 (4.9)	Farde et al. (25)
Caudate nucleus L			22.1 (5.3)		8.1 (2.0)		
Putamen R			27.6 (8.0)	23	9.1 (2.3)		
Caudate nucleus R			22.5 (7.7)		7.7 (2.4)		
Putamen	14	TEM	29.6 (6.8)	10	11.9 (3.6)	59.2 (7.4)	Rinne et al. (26)
Caudate nucleus			26.9 (7.5)		11.0 (3.2)		
Striatum R	8	TEM	28.7 (4.9)	n/a	8.2 (1.1)	36.9 (6.4)	Hietela et al. (27)
Striatum L			28.4 (5.7)		9 (2.2)		
Striatum R (males)	33	TEM	26.7 (7.6)	n/a	9.9 (2.6)	40.2 (16.7)	Pohjalainen et al. (28)
Striatum L (males)			25.4 (7.2)		9.4 (2.7)		
Striatum R (females)	21	TEM	27.2 (6.3)		10.6 (2.0)		
Striatum L (females)			27.3 (7.9)		11.0 (2.9)		
Striatum (test)	4	TEM	19.4 (2.3)	n/a	7.8 (0.9)	28.0 (7.0)	Hietela et al. (29)
Striatum (re-test, short term follow-up)			21.8 (2.2)		8.3 (0.8)		
Striatum (test)	4	TEM	28.7 (3.5)		8.6 (1.2)		
Striatum (re-test, long term follow-up)			26.3 (2.3)		8.2 (0.9)		
Striatum	3	TREMBLE	26 (4.0)	n/a	9.6 (4.0)	n/a	Wong et al. (21)

From the PET studies listed in Table 1, Farde et al. (25) and Rinne et al. (26) reported the $D_{2}$/$D_{3}$ dopamine receptor density of putamen and caudate nucleus in healthy subjects obtained by application of TEM analysis and Scatchard plots. Farde et al. (25) reported 25% higher density in putamen than caudate nucleus, while the difference reported by Rinne et al. (26) was as low as 10%. In other studies of the receptor density with TEM analysis, the authors merged the subregions and reported densities in striatum that are comparable with the results of the present TEM analysis.

It was not clear from previous PET studies whether the density of $D_{2}$/$D_{3}$ receptors is higher in putamen than in caudate nucleus. Therefore, we compared the present results with data from *in vitro* autoradiography in healthy humans, presented in (Table 2). To make the comparison with *in vitro* studies possible, we converted the density per unit wet weight to density per unit dry weight of protein. Under the assumption that brain tissue contains 10% solid material, conversion of wet to dry weight units uses division by 10 (36). The autoradiography studies listed in Table 2 support considerably higher density in putamen than in caudate nucleus. As the studies were performed *in vitro* and with other ligands of the $D_{2}$/$D_{3}$ receptors, the results are not numerically translatable. However, the comparison of the relative regional difference shows that results from TREMBLE are consistent with data obtained by autoradiography.

**Table 2:** Comparison of $B_{\max}$ with autoradiography studies in healthy human brain.

Ligand		Brain region	n	$B_{\max}$ (fmol/mg protein) Mean (SD)	% Diff	Age Mean (SD)	Reference
PET	[11C]raclopride	Putamen	18	267 (119)	25	34.9 (14.0)	This study
		Caudate nucleus		180 (89)			
Autoradiography	[3H]raclopride	Putamen	20	249 (112)	14	77.2 (11.0)	Piggott et al. (30)
		Caudate nucleus		218 (98)			
	[3H]spiroperidol	Putamen	4	450 (6)	7	50.2 (14.5)	Joyce et al. (31)
		Caudate nucleus		420 (11)			
		Putamen	10	268 (28)	37	n/a	Camps et al. (32)
		Caudate nucleus		195 (30)			
	[125I]epidepride	Putamen	4	152 (25)	20	n/a	Joyce et al. (33)
		Caudate nucleus	5	127 (18)			
		Putamen^a^	24	174 (19)	30	68.0 (14.0)	Murray et al. (34)
		Caudate nucleus^a^		134 (21)			
		Putamen	4	100^b^ (2.7)	22	n/a	Hall et al. (35)
		Caudate nucleus		82^b^ (0.3)			

The studies listed in the table consistently showed that putamen has markedly higher receptor density than caudate nucleus. %Diff denotes the percentage difference in $B_{\max}$ between putamen and caudate nucleus.

^a^Indicates rostral part of striatum.

^b^Indicates the mean % of the density in putamen.

We also compared the values of $BP_{\text{ND}}$ from the TEM and TREMBLE analyses of this study with the results reported in the literature. The Eadie-Hofstee regression is driven by the magnitudes of $BP_{\text{ND}}$ and the bound quantity of tracer ($M_{b}$ or $M_{b}^{*}$, depending on method). Table 3 lists the publications that included values of $BP_{\text{ND}}$ obtained by linear regression methods, including the reference region version of the Logan plot (42) and the simplified reference tissue method (SRTM) (43). The publications listed in Table 3 consistently report greater values of $BP_{\text{ND}}$ in putamen than in caudate nucleus, with a difference of 20–30% that matches the results of TREMBLE. The lack of a difference between estimates of $BP_{\text{ND}}$ for the two striatal regions with TEM analysis can be attributed to the use of cerebellum activity in TEM as an approximation of non-specifically bound ligand in regions of binding. Instead, the correct estimate of unbound ligand in a region of specific binding depends on the number of receptors available. When receptors are blocked, the quantity of unbound ligand increases as the receptor availability declines. Thus, the use of cerebellum activity as an estimate of unbound and non-displaceable ligand in the computation of specific binding is erroneous. Ito and colleagues (44) simulated a study to characterise the error associated with the use of cerebellum activity in TEM instead of the real plasma input function. The authors demonstrated that TEM caused larger errors and led to overestimation of the values of $BP_{\text{ND}}$ when the assumed magnitudes of $BP_{\text{ND}}$ and $K_{1}$ both were low, and the authors also demonstrated mathematically that the concentration in cerebellum equals the free unbound and non-specifically bound ligand at the time of steady-state, but $dm_{\text{b}}^{*}/dt$ is not equal to zero and the bound ligand therefore is not at equilibrium at the time of steady-state identified by the TEM analysis.

**Table 3:** Comparison of $BP_{\mathrm{ND}}$ of [C11] raclopride binding in healthy human subjects.

Brain region	n	Kinetic model	$BP_{\mathrm{ND}}$ (pmol/${\mathrm{cm}}^{3}$) Mean (SD)	% Diff	Age Mean (SD)	Reference
Putamen	18	TEM	2.9 (0.3)	4	34.9 (14.0)	This study
Caudate nucleus		TEM	2.8 (0.3)			
Putamen		TREMBLE	2.0 (0.2)	25		
Caudate nucleus		TREMBLE	1.6 (0.3)			
Putamen (right)	8	Logan	2.1 (0.3)	24	25.0 (5.8)	Yoder et al. (37)
Caudate nucleus (right)			1.7 (0.2)			
Putamen (left)			2.3 (0.3)	41		
Caudate nucleus (left)			1.6 (0.3)			
Putamen	110	Logan	3.0 (0.4)	30	n/a	Zhou et al. (38)
Caudate nucleus			2.3 (0.3)			
Putamen	10	Logan	2.8 (0.4)	33	32.2 (11.1)	Black et al. (39)
Caudate nucleus			2.1 (0.5)			
Putamen	12	Logan	2.6 (0.2)	13	63.0 (7.0)	Politis et al. (40)
Caudate nucleus			2.3 (0.2)			
Putamen	10	SRTM	3.0 (0.3)	36	n/a	Shotbolt et al. (41)
Caudate nucleus			2.2 (0.3)			

Logan indicates graphical anlysis using Logan plot with cerebellum as reference input. SRTM indicates simplified reference tissue model. %Diff denotes the percentage difference in $BP_{\mathrm{ND}}$ between putamen and caudate nucleus.

Notably, the apparent values of $BP_{\text{ND}}$ computed with TREMBLE in the present study (Figures 5A and B) approached a constant value at the time when $dm_{\text{b}}/dt=0$. The prolonged constant ratio between bound and free ligand quantities with TREMBLE analysis strongly supports the contention that TREMBLE identifies the ultimately transient equilibrium that persisted for some time after bolus injection. Determination of $BP_{\text{ND}}$ at equilibrium is crucial to obtain valid estimates of the binding potential that is defined only at instances of true equilibrium (17). Thus, identification of the time of equilibrium is critical to accurate and reproducible quantification of the values of $B_{\text{max}}$ and $K_{\text{D}}$.

In conclusion, we demonstrated that the TREMBLE method yields valid estimates of receptor-ligand interaction at true (albeit transient) equilibrium after bolus injection of tracer. This conclusion would also apply to to experiments based on bolus-plus-infusion of the tracer, The reason is that the bolus-plus-infusion experiments focus on the establishment of constant concentrations of all tracer molecules in the tissue, rather than on the requirement of constant levels of bound tracer associated with true equilibrium, a goal that is very difficult to reach. A disadvantage of TREMBLE is the requirement for arterial plasma concentration sampling that makes clinical studies labor intensive, but the approach is important to the detection of moderate changes as found especially in neuropsychiatric disorders, for example.

## Key points

QUESTION: A true (albeit often transient) equilibrium of the binding of radioligands to neuroreceptors must be present for binding parameters to be valid. To idnetify instances of true equilibrium, we designed a method of quantitation of radioligand binding that allowed us to identify instances of transient but true equilibrium of binding.

PERTINENT FINDINGS: We compared the results of the new method of identification of instances of transient equilibrium (TREMBLE) of $D_{2}$/$D_{3}$ receptor binding of labeled raclopride in striatum of humans with the standard transient equilibrium method (TEM) that fails to identify such instances of true but transient equilibrium (TEM).

IMPLICATIONS FOR PATIENT CARE: The method of TREMBLE allows measurements of parameters of receptor binding to reach a level of accuracy that assures more precise and therefore relevant estimates of binding potentials on which to base the therapeutic engagement of dopamine receptors during treatment of pathological conditions involving dopaminergic neurotranismission of human brain.

## Figures and Tables

**Figure A1:**
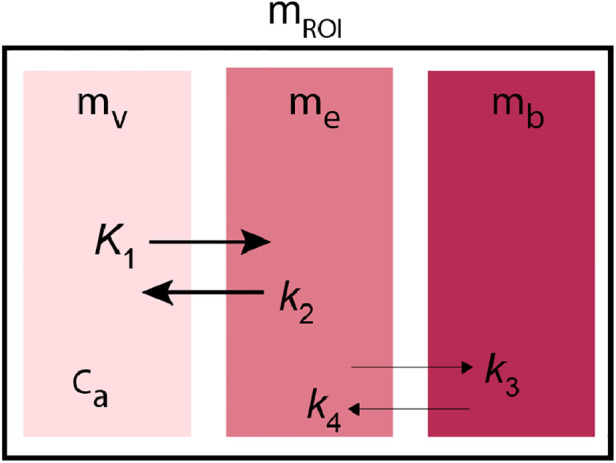
Compartment model of TREMBLE.

**Figure A2:**
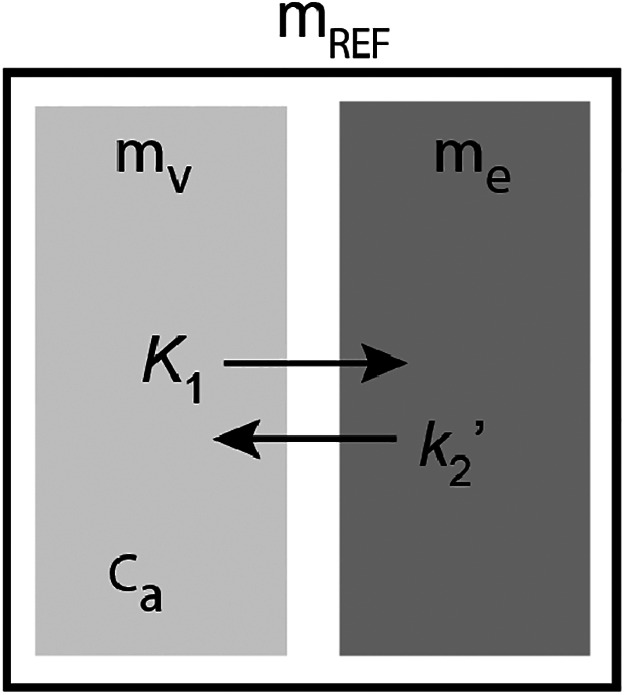
$V_{e}$ solved from a two-compartment model applied to cerebellum.

**Figure A3:**
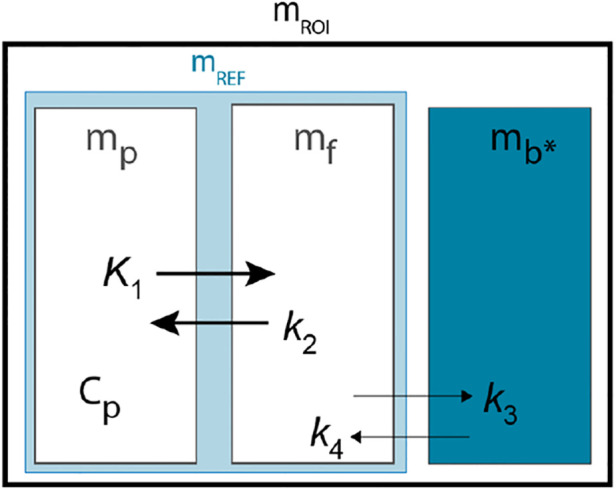
Compartment model of TEM Modified from Farde et al. (22).

## Data Availability

The datasets presented in this article are not readily available because the data is a few years old and has only the TAC curves and not the original images. Requests to access the datasets should be directed to Dean F. Wong dfwong@wustl.edu and Albert Gjedde gjedde@sund.ku.dk.

## Appendix: Theories of transient equilibrium approach

### Clarification on chosen nomenclature for binding potential equations

The nomenclature used here is based on equations reported by Gjedde et al. (45), Innis et al. (18), and Gjedde & Wong (46). However, the first-order differential equations reported by Gjedde et al. (45) and Gjedde & Wong (46) are not included in the formalism presented by Innis et al. (18) that focused on steady-state solutions. The first-order differential equations are shown below as the equivalent basis for the transient solutions on which TREMBLE depends. The steady-state solutions reported by Innis et al. (18) are equivalent to the steady-state solutions reported by Gjedde et al. (45) and will be presented as such at the appropriate place in the Appendix. The main distinction concerns the difference between the units of mass per unit volume used by Gjedde et al. (45) and Gjedde & Wong (46), and the units of concentration used by Innis et al. (18) that refer to spaces of tissue rather than volumes of fluid. It is the contention of Phan et al. authors that the units of mass per unit volume are more appropriate. In the retrospect we will suggest that the Innis et al (18) be modified in future extensions of nomenclature of Innis et al. (18).

### I. TRansient EquilibriuM BoLus Estimation

The TRansient EquilibriuM Bolus Estimation (TREMBLE) method estimates the quantity of bound ligand to receptors when an equilibrium of bound and unbound tracer quantities is assumed to occur transiently. The method was introduced by Sølling et al. (20) and Wong et al. (21). As illustrated in Figure A1, the kinetic model describes two simultaneous dynamics, the tracer transfer from the plasma compartment ($m_{\text{v}}$) to the exchangeable tissue compartment ($m_{\text{e}}$) and to a separate compartment of specifically bound tracer ($m_{\text{b}}$). The first order differential equation describes the distribution in the vascular compartment,

(A1) $$\frac{dm_{v}(t)}{dt}=V_{0}\;\frac{dc_{\text{a}}(t)}{dt},$$

where $m_{b}$ is the mass of ligand detectable by the tomograph and $V_{0}$ denotes the volume of the vascular bed, and $c_{\text{a}}(t)$ is the time-variable concentration in plasma. The exchange from plasma to the exchangeable compartment can be described as follows,

(A2) $$\frac{dm_{e}(t)}{dt}=K_{1}c_{\text{a}}(t)-k_{2}m_{e}(t)+k_{4}m_{b}(t)-k_{3}m_{e}(t),$$

and to the binding compartment as follows,

(A3) $$\frac{dm_{b}(t)}{dt}=k_{3}m_{e}(t)-k_{4}m_{b}(t),$$

where $K_{1}$ denotes the plasma clearance, $k_{2}$ is the rate constant of exchange from tissue to plasma, and $m_{e}$ and $m_{b}$ denote the respective quantities in the exchangeable and specific bound compartments.

Multilinear analysis can be applied to fit multiple unknown variables, but with three or more compartments the number of unknown parameters and degrees of freedom become excessive to the extent that the analysis fails to yield accurate rate constants. To avoid the uncertainty of many variables, the fundamental aim of the analysis is to identify the quantity in an intermediate compartment $m_{\text{e}}$ that controls the exchange of ligands with subsequent compartments. Only in the initial phase of an tomography session is the quantity of ligand bound to receptors is negligible, and only then is the flux of ligand is controlled by the concentration gradient between plasma and brain tissue. At all time, the measured activity in a region of interest $\left(m_{\text{ROI}}\right)$ obeys the equation,

(A4) $$\frac{dm_{\text{ROI}}}{dt}=V_{0}\frac{dc_{\text{a}}(t)}{dt}+K_{1}c_{\text{a}}(t)-k_{2}m_{e}(t),$$

such that rearrangement of Eq. A4 yields the quantity of ligand in the exchangeable compartment,

(A5) $$m_{\text{e}}(t)=V_{\text{e}}\left[c_{\text{a}}(t)-\frac{1}{K_{1}}\left(\frac{dm_{\text{ROI}}(t)}{dt}-V_{0}\frac{dc_{\text{a}}(t)}{dt}\right)\right],$$

where $V_{\text{e}}$ is the partition volume that equals $K_{1}/k_{2}$. To solve Eq. A5, it is necessary to estimate $V_{0}$, $K_{1}$ and the derivatives of $c_{\text{a}}(t)$ and $m_{\text{ROI}}(t)$. The estimation of $V_{0}$ and $K_{1}$ using the Gjedde-Patlak plot and estimation of $V_{\text{e}}$ in a reference region with the two-compartment model is shown in separate sections below. When the quantity of ligand in the exchangeable compartment is known, the bound quantity is obtained by subtraction,

(A6) $$m_{\text{b}}(t)=m_{\text{ROI}}(t)-m_{v}(t)-m_{\text{e}}(t).$$

where at transient equilibrium, the first derivative of the bound quantity, $dm_{\text{b}}(t)/dt$, equals zero. Innis et al. (18) correctly cite Mintun et al. (17) for the definition of binding potential as “the equilibrium ratio of specifically bound ligand (B) to its free concentration (F),” which we here interpret as the quantities of bound and unbound ligand at equilibrium, as expressed in Eq. A7 below. The time of the transient equilibrium corresponds to a peak of the curve of bound ligand, and at this time the steady-state binding potential is defined as the ratio of bound to free ligand, indicated by upper case symbols, $M_{b}$ and $M_{e}$,

(A7) $$BP_{\mathrm{ND}\;(\mathrm{TREMBLE})}=\frac{M_{\text{b}}}{M_{\text{e}}}.$$

#### Estimation of $V_{e}$ from a reference region

Assuming that the volume of distribution, equal to the ratio of the rate constants $K_{1}$ and $k_{2}$, is identical in a reference region and in the regions of specific binding, the ratio of $K_{1}$ to $k_{2}$ in cerebellum can be obtained from the radioactivity in plasma and cerebellum.

When the specific binding of the ligand is negligible in cerebellum, as assumed, the kinetic model of three compartments is reduced to two compartments, as illustrated in Figure A2. The operational equation is then,

(A8) $$\frac{dm_{\mathrm{REF}}}{dt}=\frac{dm_{v}(t)}{dt}+\frac{dm_{e}(t)}{dt}=V_{0}\frac{dc_{\text{a}}(t)}{dt}+(K_{1}c_{\text{a}}(t)-k_{2}^{\prime }m_{e}(t)),$$

where $k_{2}^{\prime }$ refers to the magnitude of $k_{2}$ in cerebellum. Then, the total quantity of ligand in the reference region, $m_{\mathrm{REF}}$ is obtained by integration of the differential Eq. A8,

(A9) $$m_{\mathrm{REF}}(T)=V_{0}\;C_{a}(T)+K_{1}\int_{0}^{T}c_{\text{a}}(t)\;dt-k_{2}^{\prime }\int_{0}^{T}m_{e}(t)\;dt.$$

Because $m_{e}$ is unknown, $m_{e}$ is expressed in terms of $m_{\mathrm{REF}}$, and substitution of $m_{e}$ with the expression $m_{e}=m_{\mathrm{REF}}(1-m_{\text{v}})/m_{\mathrm{REF}}$ yields,

(A10) $$m_{\mathrm{REF}}(T)=V_{0}C_{a}(T)+K_{1}\int_{0}^{T}c_{\text{a}}(t)\;dt-k_{2}^{\prime }\int_{0}^{T}m_{\mathrm{REF}}(t)\left(1-\frac{m_{\text{v}}(t)}{m_{\mathrm{REF}}(t)}\right)dt$$

At later times, the approaching equilibrium state is associated with substantial washout of ligand from plasma, and the ligand accumulates in brain tissue and other organs. At this time, the magnitudes of $C_{a}(T)$ and the ratio $m_{\text{v}}/m_{\text{REF}}$ become negligible. Thus, Eq. A10 reduces to,

(A11) $$m_{\mathrm{REF}}(T)=K_{1}\int_{0}^{T}c_{\text{a}}(t)\;dt-k_{2}^{\prime }\int_{0}^{T}m_{\mathrm{REF}}(t)\;dt.$$

where division of Eq. A11 with $\int_{0}^{T}c_{\text{a}}(t)\;dt$ yields a simple linearized solution as previously described by Gjedde et al. (47),

(A12) $$\frac{m_{\mathrm{REF}}(T)}{\int_{0}^{T}c_{\text{a}}(t)\;dt}=K_{1}-k_{2}^{\prime }\frac{\int_{0}^{T}m_{\mathrm{REF}}(T)\;dt}{\int_{0}^{T}c_{\text{a}}(t)\;dt}.$$

We resolved the cerebellar rate constants $K_{1}$ and $k_{2}^{\prime }$ graphically from the y-intercept and slope, respectively, and $V_{\text{e}}$ is simply the the ratio of the cerebellar $K_{1}$ and $k_{2}^{\prime }$.

#### Estimation of the vascular volume $V_{0}$

Regional $V_{0}$ and $K_{1}$ values can be estimated by means of the two-compartment model applied to the estimation of $V_{\text{e}}$. At the onset of the PET acquisition, the tracer predominantly exists in the vascular compartment, and the loss of tracer from brain to plasma can be ignored,

(A13) $$m_{\mathrm{ROI}}(T)=V_{0}c_{\text{a}}(t)+K_{1}\int_{0}^{T}c_{\text{a}}(t)\;dt,$$

where $M_{\mathrm{ROI}}(T)$ is the observed radioactivity in a region of interest. When divided by $c_{\text{a}}(t)$, Eq. A13 yields the equation of Gjedde-Patlak plot (47,48),

(A14) $$\frac{m_{\mathrm{ROI}}(T)}{c_{\text{a}}(t)}=V_{0}+K_{1}\frac{\int_{0}^{T}c_{\text{a}}(t)\;dt}{c_{\text{a}}(t)},$$

where $V_{0}$ is solved from the y-intercept, and the value of $K_{1}$ is obtained from the initial acquisition frames. In this study, we solved regional $V_{0}$ and $K_{1}$ at 0-2 minutes post-injection.

### II. Transient equilibrium model

The ligand and neuroreceptor interaction described by Farde and colleagues (22) as the Transient Equilibrium Model (TEM) method (22) also involves a three-compartment model and four first order rate constants (Figure A3). In contrast to TREMBLE, however, where the center compartment represents the quantity of exchangeable ligand, the center compartment of TEM represents unbound or “free” quantity of the tracer denoted as $m_{f}$.

The exchange of ligand in the compartments can be described by two differential equations,

(A15) $$\frac{dm_{f}(t)}{dt}=K_{1}C_{p}(t)-k_{2}m_{\text{\textbackslash{},f}}(t)+k_{4}(t)m_{\text{b}}^{*}(t)-k_{3}m_{\text{\textbackslash{},f}}(t)$$

and

(A16) $$\frac{dm_{b}(t)}{dt}=k_{3}m_{\text{\textbackslash{},f}}(t)-k_{4}(t)m_{\text{b}}^{*}(t),$$

where $m_{\text{\textbackslash{},f}}$ denotes the free quantity in tissue and $C_{\text{\textbackslash{},p}}$ is the concentration in plasma. For convenience, the specifically bound quantity in tissue alleged by TEM is denoted with an asterisk ($m_{\text{b}}^{*}$). Assuming that specific binding of [C11] raclopride is negligible in the reference region, the exchange of ligand in the reference region is reduced to,

(A17) $$\frac{dm_{\text{REF}}(t)}{dt}=K_{1}C_{\text{\textbackslash{},p}}(t)-k_{2}m_{\text{\textbackslash{},f}}(t)$$

and

(A18) $$dm_{\text{ROI}}(t)=dm_{\text{REF}}(t)+dm_{\text{b}}^{*}(t).$$

where by rearrangement of Eq. A18, the specifically bound quantity yields,

(A19) $$m_{\text{b}}^{*}(t)=m_{\text{ROI}}(t)-m_{\text{REF}}(t).$$

In TEM, the alleged specifically bound quantity $m_{\text{b}}^{*}$ is obtained by subtraction of the quantity in the reference region ($m_{\mathrm{ref}}$) from the total quantity ($m_{\mathrm{ROI}}$). In this calculation, it is assumed that the free quantities in tissue and in plasma have equal magnitudes in $m_{REF}$ and $m_{\mathrm{ROI}}$. As in TREMBLE, quantity of specifically bound ligand then is obtained in TEM at the time of transient equilibrium when the first derivative $dm_{\text{b}}^{*}(t)/dt$ equals zero. The equilibrium binding potential in TEM is determined from the ratio of the bound quantity in a region of binding and the total quantity of tracer in the reference region,

(A20) $$BP_{\mathrm{ND}\;(\mathrm{TEM})}=\frac{M_{b}^{*}}{M_{REF}}.$$

where it is important to note that the binding potential obtained by TREMBLE in principle therefore is different from the binding potential obtained by TEM because assumptions of non-displaceable quantities have different origins and are determined differently.

### III. Inhibition plots of receptor saturation

We used Inhibition Plot procedures (49) (see Figure A4 below) to determine the degree of competition between labeled and unlabeled tracer molecules, to rule out degrees of competition that would be inconsistent with active exchange of labeled ligand molecules, because of complete or close-to-complete saturationffo receptors (Figure A4 and Table A1).

**Table A1:** Regional $V_{T}$ values of the three excluded subjects.

	Ex 1			Ex 2			Ex3		
ROI	$V_{T(b)}$	$V_{T(i)}$	$\Delta V_{T}$	$V_{T(b)}$	$V_{T(i)}$	$\Delta V_{T}$	$V_{T(b)}$	$V_{T(i)}$	$\Delta V_{T}$
Putamen	1.25	0.58	0.67	1.15	0.42	0.73	1.225	0.367	0.858
Putamen left	1.23	0.58	0.65	1.21	0.45	0.76	1.211	0.339	0.872
Putamen right	1.22	0.58	0.64	1.10	0.39	0.71	1.239	0.388	0.851
Caudate nucleus	0.95	0.53	0.42	1.00	0.32	0.68	1.109	0.272	0.837
Caudate nucleus left	0.84	0.51	0.33	0.93	0.29	0.64	1.117	0.295	0.822
Caudate nucleus right	1.08	0.56	0.52	1.04	0.36	0.68	1.065	0.243	0.822
Cerebellum	0.34	0.35	−0.01	0.34	0.35	−0.01	0.321	0.317	0.004

The $V_{\text{T}}$ values in the three excluded subjects are listed. $V_{\text{T(b)}}$ and $V_{\text{T(i)}}$ denote the distribution volume at baseline and inhibition (challenge) conditions, whereas $\Delta V_{\text{T}}$ indicates the difference in $V_{\text{T}}$ between the two conditions. The $V_{\text{T}}$ estimates are obtained from graphical analysis as previously described by Phan et al. (50).

### IV. Eadie-Hofstee plots of receptor density and affinity

We jointly determined receptor density and affinity by graphical means of the Eadie-Hofstee plot (55), based on binding parameters obtained at two different levels of receptor occupancy. The steady-state estimates of $BP_{\text{ND}}$ were plotted as a function of the bound quantity (B) (Figure 4),

(A21) $$B=B_{\text{max}}-(V_{\text{e}}K_{\text{D}})BP_{\text{ND}},$$

where the ordinate intercept yielded the receptor density $B_{\text{max}}$, and the slope yielded the half-saturation concentration, equal to the reciprocal of the affinity $1/K_{\text{D}}$. Innis et al. (18) focus on the number of free receptor sites ($B_{\mathrm{avail}}$) that equals the the difference between the total number of sites and the sites holding bound ligand(s). It is possible to rephrase the equation with the term $B_{\mathrm{avail}}$ by substitution, such that the Eadie-Hofstee equation at equilibrium now reads

(A22) $$B_{\text{max}}-B_{\mathrm{avail}}=B_{\text{max}}-(V_{\text{e}}K_{\text{D}})BP_{\text{ND}},$$

or

(A23) $$B_{\mathrm{avail}}=(V_{\text{e}}K_{\text{D}})BP_{\text{ND}},$$

that allows the calculation of the Michaelis constant with unit of concentration from values of $B_{\mathrm{avail}}$, $V_{\text{e}}$, and $BP_{\text{ND}}$.

### V. Radiochemistry Details in Low Molar Activity Scans

In low molar activity scans, nonradioactive raclopride in sterile saline solution was added to the [C11] raclopride sterile saline solution. The solution was prepared just prior to injection into the subject. The final molar activity adjusted to the injection adjusted to the injection time was used for the calculation of $B_{\text{max}}$ and $K_{\text{D}}$. The molar activity at baseline (high molar activity) was always higher than the challenge (low molar activity). The individual values are listed in Table A2.

**Table A2:** Individual molar activity.

Subject	High molar activity (HMA)	Low molar activity (LMA)	HMA/LMA
	116.09	0.86	135
	411.01	0.61	678
	44.57	0.65	69
	924.87	0.73	1259
	1480.97	0.68	2181
	271.77	0.75	364
	706.78	0.43	1651
Ex 1	35.85	0.42	86
	140.69	0.74	189
	272.27	0.72	377
Ex 2	32.20	0.71	45
	533.36	0.61	873
	1424.43	0.95	1498
	75.58	0.84	90
	53.98	0.56	96
	90.96	0.72	127
Ex 3	83.74	0.63	134
	76.17	0.48	158
	228.12	0.61	373
	282.89	0.80	355
	254.32	0.60	425
Mean	359.08	0.67	
SD	432.14	0.14	

The values of individual high and low molar activities (HMA and LMA) are listed in units of GBq/μmol. The values in the three excluded subjects are indicated as Ex 1,2 and 3.
